# Combined Application of Chitosan-Induced *Wickerhamomyces anomalus* and *Bacillus subtilis* to Control Blue Mold Disease of Table Grapes

**DOI:** 10.3390/foods15101630

**Published:** 2026-05-07

**Authors:** Esa Abiso Godana, Gerefa Sefu Edo, Habiba Lawal, Aasia Muhammed Jamiu, Kaili Wang, Qiya Yang, Asanda Mditshwa, Hongyin Zhang

**Affiliations:** 1 School of Food and Biological Engineering, Jiangsu University, Zhenjiang 212013, China; esaabiso@gmail.com (E.A.G.); sefgare@gmail.com (G.S.E.); habeebalawal1@gmail.com (H.L.); hasiatemjay@gmail.com (A.M.J.); 1000005483@ujs.edu.cn (K.W.); yangqiya1118@163.com (Q.Y.); 2 Department of Horticultural Science, School of Agricultural Earth & Environmental Science, University of KwaZulu Natal, Private Bag X01, Scottsville, Pietermaritzburg 3209, South Africa

**Keywords:** postharvest biocontrol, *Penicillium expansum*, table grapes, *Bacillus subtilis*, *Wickerhamomyces anomalus*, chitosan, synergistic combination

## Abstract

Blue mold decay caused by *Penicillium expansum* is a major disease of table grapes, causing substantial economic losses. With increasing environmental concern and consumer interest in chemical-free products, eco-friendly biocontrol strategies are necessary. The strains of *Wickerhamomyces anomalus* incubated with chitosan (WaC) and *Bacillus subtilis* (Bs) are already reported for their promising efficiency to control blue mold disease caused by *P. expansum*. However, their individual application is less effective compared to chemical fungicides. The current study determined combined application of these two prominent antagonistic microbes against blue mold decay of grapes. Initially we carried out a compatibility study which confirmed that there was no mutual inhibition among the strains, enabling synergistic formulations. An in vitro study showed that the combined application of WaC and Bs better reduces *P. expansum* colony diameters at a 4:1 ratio. This ratio also maximally suppressed the pathogen spore germination (62% reduction) and germ tube elongation (67% reduction). The in vivo studies also showed that WaC + Bs (at 4:1) significantly minimized lesion diameters and decay rates of grapes during the 13-day storage, outperforming individual applications. The physicochemical quality parameters of table grapes were also better preserved. The treatments modulated antioxidant and defense enzymatic activities such as PPO, POD, PAL, APX, SOD, and CHI. The elevated phenolic and flavonoid contents, with strong positive correlations among the enzymes, underscored the induced resistance of the host. These findings highlight the synergistic potential of chitosan-enhanced Wa with Bs for effective, sustainable postharvest biocontrol, extending grape shelf life and reducing chemical reliance.

## 1. Introduction

Table grapes (*Vitis vinifera* L.) are among the most highly valued fruits worldwide because of their taste and nutritional content, such as high levels of antioxidants, vitamins, and bioactive compounds [1]. The International Organization for Vine and Wine (OIV) estimated that, in the year 2024 alone, around 77.7 million tons of fresh grapes are produced in the world, and China is among the leading producers (17.0 million metric tons), followed by Italy (7.3 million tons) and the USA (6.4 million metric tons). Spain, France, India, Turkey, South Africa, Argentina, and Chile are among the large producers of fresh grapes globally. These countries export fresh grapes worldwide, with China alone exporting around 594 kilotons, followed by Peru (554 kilotons) and Chile (523 kilotons).

Despite this large volume of fruit production, postharvest decay poses a major challenge, leading to substantial economic losses estimated to 20–50% every year [2]. Blue mold decay caused by *Penicillium expansum* is among the major causes of the decay. Despite the fruit deterioration, this pathogen also produces patulin, a mycotoxin harmful to human and animal health [3]. Postharvest conditions such as high humidity and ambient temperature favor the invasion of this pathogen. Chemical fungicides such as thiabendazole and imazalil have been the first choice in the past [4]. The concern about environmental effects, fear of fungal resistance to chemicals, residual concern, and increasing consumer interest toward chemical-free products have led to a search for alternative methods [5]. Therefore, the need for sustainable alternatives has shifted focus toward biocontrol strategies, which utilize antagonistic microorganisms to inhibit pathogen growth without compromising fruit quality or safety.

In our previous studies, we identified a strain of *Wickerhamomyces anomalus* (formerly known as *Pichia anomala*) as a promising biocontrol agent that inhibits blue mold disease of grapes [6]. The yeast showed high competition for nutrient and space with the pathogen, produced volatile organic compounds (VOCs), and induced the disease defense ability of table grapes. The efficiency of this strain was even better when incubated with 1% chitosan. Different strains of *W. anomalus* are reported for their high efficiency in controlling postharvest diseases of different fruits and vegetables [7].

Enhancing the efficacy of antagonists with natural elicitors such as chitosan, trehalose, phytic acid, alginate oligosaccharides, etc., has further improved the biocontrol outcome of different yeast strains [8]. However, compared to chemical pesticides, their efficiency is still low, which affects the commercial market share of biocontrol agents. Therefore, the combined application of promising antagonists might have a synergetic effect compared to using them alone and better alter the fruit surface microbiota. With this in mind, the current study determined the effect of the combined application of *W. anomalus* incubated with chitosan and *Bacillus subtilis* to control blue mold disease of grapes.

*B. subtilis* is well known for its antagonistic properties and can offer complementary pathogen inhibition against *P. expansum* together with *W. anomalus*. The production of lipopeptides such as surfactins, iturins, and fengycins by *B. subtilis* are among the major mechanisms that disrupt fungal cell membranes and inhibit spore germination [9]. Therefore, the yeast–bacteria consortia better create diverse modes of action to achieve superior pathogen suppression. The other bacterial antagonist *Serratia liquefaciens* was also registered as a potential biocontrol agent of postharvest diseases in Google patent (WO1993018654A1) in 1993 (https://patents.google.com/patent/WO1993018654A1/en) (accessed on 28 April 2026). The strain was proposed as potential biocontrol agent against *Alternaria brassicicola* and/or *Botrvtis cinerea* on cabbage leaves.

With this in mind, the current study aimed to evaluate the antagonistic potential of *B. subtilis*, chitosan-enhanced *W. anomalus*, and their combinations against *P. expansum* both in vitro and in vivo. We determined the strains’ compatibility, their fungal inhibition, storage impacts on decay and postharvest quality parameters, enzymatic modulations, gene expression, and phenolic/flavonoid accumulation of table grapes. The correlation between the antifungal compounds and disease defense-related enzymes was also studied to further examine the control mechanisms.

## 2. Materials and Methods

### 2.1. Yeast (Wickerhamomyces anomalus)

The antagonistic yeast *W. anomalus* strain TL0903 was formerly isolated from chemical-unsprayed soil found near Zhenjiang city in Jiangsu Province, China, and was preserved in our laboratory in 70% glycerol at −80 °C. The strain was activated twice in nutrient yeast dextrose broth (NYDB) and preserved in nutrient yeast dextrose agar (NYDA) for the subsequent experiments. For each experiment, the yeast was reactivated in NYDB (cultured in a 250 mL Erlenmeyer flask containing 50 mL NYDB for 24 h), and then the cells were centrifuged at 8000× *g* for 4 min and washed two times with double distilled water to remove the growth medium. A hemocytometer (XB-K-250, Jianling Medical Device Co., Danyang, China) was used to determine and adjust the required concentration.

### 2.2. Bacteria (Bacillus subtilis and Serratia liquefaciens)

The strains of *B. subtilis* and *S. liquefaciens* recently isolated and preserved in our lab at −80 °C by our research team were used for the current experiment. For each experiment, the bacteria were reactivated in LB (Luria Broth) medium (cultured in a 250 mL Erlenmeyer flask containing 50 mL LB for 24 h), and then the cells were centrifuged by Eppendorf 5804 (Eppendorf SE, Hamburg, Germany) at 8000× *g* for 4 min and washed two times with double distilled water to remove the growth medium. A hemocytometer XB-K-250 (Jianling Medical Device Co., Danyang, China) was used to determine and adjust the required concentration.

### 2.3. Pathogen (Penicillium expansum)

*P. expansum* was previously isolated from infected grapes by our research team, and the strain was preserved in our laboratory at −80 °C. Prior to use, the strain was activated twice in potato dextrose broth (PDB) and then preserved in potato dextrose agar (PDA) for the subsequent experiments. Before each experiment, PDA was used to induce a fresh culture of *P. expansum*. After 7 days of growth at 25 °C, spore suspension was prepared by removing the spores with a bacterial loop and suspending them in double distilled water. The dilution was thoroughly mixed for around 30 min by using a vortex, and the suspension was then adjusted to the required concentration using hemocytometer XB-K-250 (Jianling Medical Device Co., Danyang, China).

### 2.4. Affinity Test Between the Yeast and Bacterial Strains

The yeast and bacterial strains were prepared as mentioned in Section 2.2 and Section 2.3, respectively. The strains Wa (*W. anomalus* alone), Wa+C (*W. anomalus* incubated with chitosan), Bs (*B. subtilis*), and Sl (*S. liquefaciens*) were two by two cross hatched on the PDA medium. The strains were incubated at 28 °C for 3 days and their growth condition on the medium was carefully observed. The strains were considered antagonistic to each other if there was no growth at the intersection point where the two strains met during the cross hatching. However, if the two strains kept growing at the intersection as normal, they were considered as positive affinity and could be combined to form a composite biocontrol agent [10].

### 2.5. Grapes

Fresh grapes (*V. vinifera* cv. Hongti) were obtained from a vineyard found near Zhenjiang City, Jiangsu Province, in China. Healthy fruits, free from chemical residues, with no mechanical damage and with uniform maturity, color, and size, were carefully chosen. The fruits were first washed with tap water, then soaked in 0.1% sodium hypochlorite solution for 4–5 min and finally rinsed again with tap water. Then, the fruits were dried at room temperature for around 2 to 3 h and got ready for the subsequent experiments.

### 2.6. Determination of the Best Combination and Ratio with Inhibitory Effect on Penicillium expansum In Vitro and In Vivo

Since we confirmed that all the strains can grow together without inhibiting each other during the affinity test experiments, we then determined the best combination at four ratio levels against their inhibitory effect on *P. expansum* both in vitro and in vivo. The combinations were as follows: Bs:Wa, Bs:WaC, Sl:Bs, Sl:Wa, Sl:WaC, and Wa:WaC, all of them at ratios of 1:1, 1:2, 1:3, 1:4, 4:1, 3:1, and 2:1. The bacterial and yeast concentration was 1 × 10^8^ cells/mL, while that of the pathogen was 1 × 10^5^ cells/mL. After the strains were prepared in the mentioned ratios, 10 µL of each mixture was injected into the holes of PDA, and after two hours, 10 µL of *P. expansum* spore suspension was added to each hole. Double distilled water was used as a control. The samples were incubated at 25 °C, and *P. expansum* lesion diameter growth was recorded after 3 days.

For the in vivo experiment, the grapes were prepared as mentioned in Section 2.1. Then, uniform wounds (2 mm diameter and 2 mm deep) were made at the center point of the berries. After the strains were prepared in the mentioned ratio, 10 µL of each mixture was injected into the prepared grape holes, and after two hours, 10 µL of *P. expansum* spore suspension was added to each hole. Double distilled water was used as a control. After drying, the samples were incubated at 25 °C and 95% RH in a plastic basket molded to contain 24 grapes individually. The growth of lesion diameter was recorded after 3 days. Three replicates were performed per treatment, and the experiments were repeated twice to obtain confirmatory results.

### 2.7. Effect of the Combined Microbes on the Spore Germination Rate and Germ Tube Length of Penicillium expansum

Both the in vitro and in vivo experiments confirmed that the combination of *B. subtilis* together with *W. anomalus* incubated with chitosan at a ratio of 1:4 better inhibits the growth of *P. expansum*. Therefore, we chose their combination at the mentioned ratio for the consecutive experiments. To determine the spore germination rate and the germ tube length, *B. subtilis* alone, *W. anomalus* incubated with chitosan, and a combination of *B. subtilis* and *W. anomalus* incubated with chitosan at a ratio of 1:4 were inoculated into 20 mL potato dextrose broth (PDB) medium separately. Double distilled water was used as a control. A spore suspension of *P. expansum* at a concentration of 1 × 10^5^ spores/mL was then inoculated to the PDB medium, and the cultures were incubated at 25 °C and 75 rpm. The germination rate and germ tube length of *P. expansum* were measured at 10 h and 12 h post-inoculation, respectively [10]. Three replicates were performed per treatment, and the experiments were repeated twice to obtain confirmatory results.

### 2.8. Effect of the Combined Microbes on the Decay Rate and Lesion Diameter of Penicillium expansum

To determine the decay rate and lesion diameter, the grapes were prepared as mentioned in Section 2.1. Then, 10 µL of a mixture of *B. subtilis* together with *W. anomalus* incubated with chitosan at a ratio of 1:4 was injected into the prepared grape holes, and after two hours, 10 µL of *P. expansum* spore suspension was added to each hole. Double distilled water was used as a control. After drying, the samples were incubated at 25 °C and 95% RH in a plastic basket molded to contain 24 grapes individually. The decay rate and lesion diameter were recorded after five days of storage. Three replicates were performed per treatment, and the experiments were repeated twice to obtain confirmatory results.

### 2.9. Determination of Natural Decay and Other Quality Parameters of Table Grapes

Grapes with uniform color, size, maturity, and free from defects were selected and divided into four groups. The first group was fully immersed in a suspension of *B. subtilis* at a concentration of 1 × 10^8^ cells/mL, the second group in *W. anomalus* incubated with chitosan at a concentration of 1 × 10^8^ cells/mL, the third group in a combination of *B. subtilis* and *W. anomalus* at the same concentration, and the fourth group in double distilled water (control). The fruits were then dried, placed in plastic crates, and stored at 20 °C. The natural decay rate and other postharvest quality parameters were determined according to the following methods.

The natural decay was determined by counting the number of decayed grapes (with visual observation). The grape decay rate was determined as: Decay rate = (Number of decayed grapes/Total number of grapes) ×100%. The weight loss was measured by digital balance MP2000-2 (±0.001 g), and the calculation was: Weight loss = [(Initial weight − Final weight)/Initial weight] × 100%. The fruit firmness was determined by a TA.XT Plus texture analyzer with a P/2 probe. The testing was set up as follows: speed 1 mm/s, penetration depth 5 mm, and force was recorded as Newton (N). A handheld refractometer was used to determine the total soluble solid content, and titratable acidity was determined by titration with NaOH using the following formula:

(1) $$\mathrm{TTA}\;(\%)=\frac{\mathrm{Molarity}\;\mathrm{of}\;\mathrm{NaOH}\;\times \;\mathrm{Volume}\;\mathrm{of}\;\mathrm{NaOH}\;(L)/2\;\times \;150.09\;g/\mathrm{mol}}{\mathrm{Volume}\;\mathrm{of}\;\mathrm{grape}\;\mathrm{juice}\;\mathrm{sample}\;(\mathrm{mL})}\;\times \;100$$

### 2.10. Determination of Enzymes Associated with Disease Resistance in Table Grapes

To determine changes in the enzymatic activities that are associated with disease resistance in table grapes, uniform wounds (2 mm diameter and 2 mm deep) were made at the center point of the berries. Then, the following treatments were applied to each hole: (1) control group (10 μL double distilled water), (2) Bs (10 μL *B. subtilis* at a concentration of 1 × 10^7^ cells/mL), (3) WaC (10 μL *W. anomalus* incubated with 1% chitosan at a concentration of 1 × 10^7^ cells/mL), and (4) Bs+WaC (10 μL *B. subtilis* plus *W. anomalus* incubated with 1% chitosan at a ratio of 1:4). After 2 h 10 μL *P. expansum* at concentration of 1 × 10^5^ cells/mL was added to each hole. The treated berries were then kept in plastic crates molded to keep 24 individual berries per crate and stored at 20 °C and 95% RH for 5 days. Changes in the enzymatic activities were determined according to the following methods.

#### 2.10.1. Polyphenol Oxidase (PPO)

The PPO activity was determined by extracting 0.5 g tissue in 2 mL 50 mM phosphate buffer (pH 7.8) containing 1% polyvinylpyrrolidone and 1.33 mM EDTA, centrifuging at 8800× *g* at 4 °C for 10 min. The enzyme extract (0.2 mL) was mixed with 2.8 mL catechol (50 mM), and absorbance increase was measured at 390 nm, with one unit as a 0.01 change per minute [11].

#### 2.10.2. Peroxidase (POD)

The POD activity was assayed by extracting 0.5 g tissue with 2 mL 50 mmol/L acetic acid–sodium acetate buffer (pH 5.5) containing 1 mmol/L polyethylene glycol and 4% polyvinylpyrrolidone, then centrifuging at 8800× *g* at 4 °C for 10 min. The enzyme extract (0.5 mL) was mixed with 3.0 mL of guaiacol (25 mmol/L) and 200 μL of H_2_O_2_ (0.5 mol/L), and the increase in absorbance was monitored at 470 nm, with one unit defined as a 0.01 change in absorbance per minute [12].

#### 2.10.3. Ascorbate Peroxidase (APX)

The APX activity was measured by homogenizing 0.5 g of tissue in 2 mL of 0.1 mol/L phosphate buffer (pH 7.5) with 2 mmol/L ascorbic acid, 5 mmol/L ethylenediaminetetraacetic acid, and 3% polyvinylpyrrolidone, followed by centrifugation at 8800× *g* at 4 °C for 10 min. The reaction mixture consisted of 0.5 mL enzyme extract and 180 μL phosphate buffer (50 mmol/L, pH 7.5 with 0.1 mmol/L EDTA and 0.5 mmol/L ascorbic acid), initiated with 20 μL H_2_O_2_ (2 mM), and absorbance decrease was recorded at 290 nm, with one unit as the oxidation of 1 μmol ascorbate per minute [13].

#### 2.10.4. Phenylalanine Ammonia-Lyase (PAL)

The PAL activity was assayed by extracting 0.5 g tissue with 2 mL 0.1 M borax–borate buffer (pH 8.8), centrifuging at 8800× *g* at 4 °C for 10 min. The extract (50 μL) was mixed with 400 μL borax–borate buffer and 50 μL L-phenylalanine, incubated at 37 °C for 60 min, stopped with 100 μL HCl, and absorbance measured at 290 nm for trans-cinnamic acid formation, with one unit as 1 nmol product per hour [14].

#### 2.10.5. Superoxide Dismutase (SOD)

The activity of SOD was determined by extracting grape tissue with 50 mmol/L phosphate buffer (pH 7.8) containing 5 mmol/L dithiothreitol and 5% polyvinylpyrrolidone, followed by centrifugation at 8800× *g* at 4 °C for 10 min. To the supernatant (0.1 mL), 1.7 mL of phosphate buffer (50 mmol/L, pH 7.8), 0.3 mL of methionine (130 mmol/L), 0.3 mL of nitro blue tetrazolium (NBT, 750 μmol/L), 0.1 mL of sodium ethylenediaminetetraacetate (100 μmol/L), and 0.3 mL of riboflavin (20 μmol/L) were added. The mixture was exposed to 4000 lx light for 15 min, and absorbance was measured at 560 nm, with one unit of SOD activity defined as the amount inhibiting NBT reduction by 50% [15].

#### 2.10.6. Chitinase (CHI)

The CHI activity was determined by extracting 0.5 g tissue in 2 mL 0.1 mol/L phosphate buffer (pH 5.7) containing 1 mM EDTA and 5 mM β-mercaptoethanol, centrifuging at 8800× *g* at 4 °C for 10 min. Phosphate buffer (580 μL) was mixed with 20 μL enzyme solution and 400 μL 1% colloidal chitin, incubated at 37 °C for 60 min, boiled for 10 min, and cooled; then 1.5 mL DNS was added, heated at 100 °C for 10 min, cooled on ice for 15 min, and absorbance measured at 540 nm [16].

### 2.11. Confirmation of Disease Resistance Related Genes Expression Levels Using RT-qPCR

The relative gene expressions of all the six enzymes associated with disease resistance of table grapes were determined according to the method described by Xu et al. [17]. The experiments were repeated two times for confirmation and there were three replications per treatment.

### 2.12. Assays of Contents of Total Phenols and Flavonoids

To determine the contents of these antifungal compounds, the table grapes were treated as described in Section 2.10. The tissue around the wound was collected and homogenized with 1% HCl–methanol in the dark. The homogenized tissue was kept at 4 °C for 20 min and afterwards centrifuged at 10,000× *g* at 4 °C for 10 min. The supernatant was used to measure the contents of total phenols and flavonoids. The total phenolic content was determined by measuring the absorbance at 280 nm, and the unit was expressed as OD280/g FW. Similarly, total flavonoid content was measured at an absorbance of 325 nm, and the unit was expressed as OD325/g FW [18].

### 2.13. Data Analysis

The collected data were subjected to analysis of variance (ANOVA) using R version 4.3.1 [19]. The mean comparisons were conducted according to Tukey’s HSD test by using the “agricolae” package in RStudio Version 1.3-7 [20] and *p* < 0.05 was considered as significantly different. The “ggplot2” package [21] was used to plot the graphs, and the “patchwork” package [22] was used to arrange the plots.

## 3. Results

### 3.1. Affinity Test Between Strains

**Figure 1:**
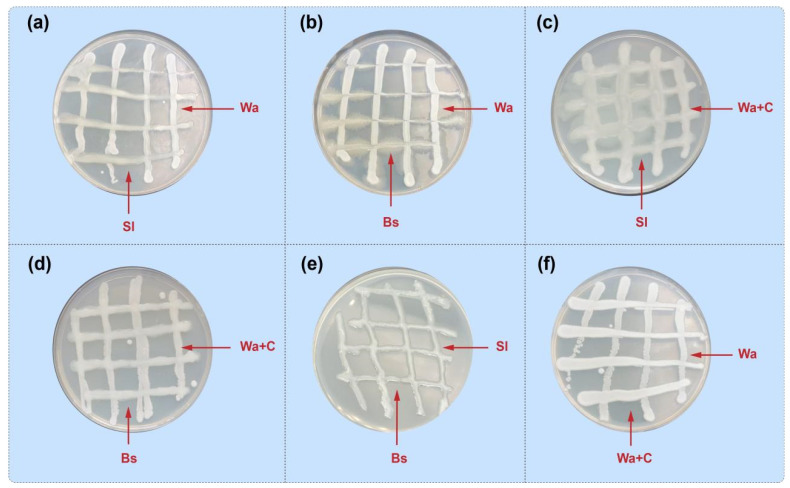
Affinity test between the bacterial and fungal antagonistic strains. *S. iquefaciens* vs. *W. anomalus* (**a**), *B. subtilis* vs. *W. anomalus* (**b**), *S. iquefaciens* vs. *W. anomalus* incubated with chitosan (**c**), *B. subtilis* vs. *W. anomalus* incubated with chitosan (**d**), *B. subtilis* vs. *S. iquefaciens* (**e**), and *W. anomalus* incubated with chitosan vs. *W. anomalus* alone (**f**). Sl (*Serratia liquefaciens*), Wa (*Wickerhamomyces anomalus*), Bs (*Bacillus subtilis*), and Wa+C (*Wickerhamomyces anomalus* cultivated with 1% chitosan). The pictures were taken after 72 h of incubation at 28 °C.

First, the compatibility experiment was conducted to evaluate potential antagonistic interactions between *B. subtilis* (Bs), *W. anomalus* (Wa), *S. liquefaciens* (Sl), and chitosan-enhanced *W. anomalus* (Wa+C) on PDA plates incubated at 28 °C for 72 h. As can be seen in Figure 1a–f, the strains exhibited full compatibility, with no visible inhibition zones or growth restrictions. Streaks of each pair grew uniformly across the plates. This absence of mutual antagonism suggests that these antagonistic agents can coexist without compromising their viability, supporting their potential use in combined formulations.

### 3.2. Determination of the Best Antagonistic Combination and Ratio to Inhibit the Growth of Penicillium expansum In Vitro

**Figure 2:**
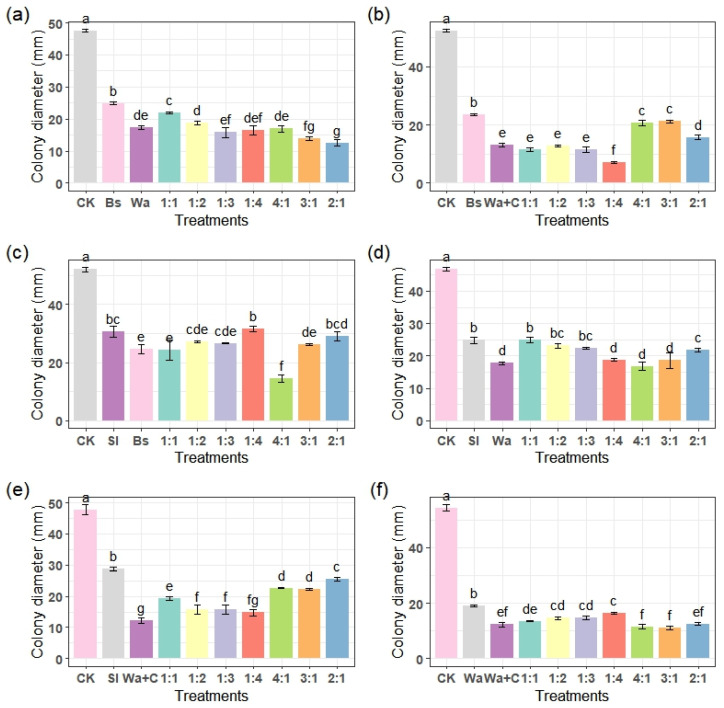
Determination of the effective combination by ratio of the antagonistic microbes that inhibit *P. expansum* growth in vitro after 4 days of storage at 28 °C. *B. subtilis* and *W. anomalus* (**a**), *B. subtilis* and *W. anomalus* incubated with chitosan (**b**), *S. iquefaciens* and *B. subtilis* (**c**), *S. iquefaciens* and *W. anomalus* (**d**), *S. iquefaciens* and *W. anomalus* incubated with chitosan (**e**), *W. anomalus* alone and *W. anomalus* incubated with chitosan (**f**). CK is double distilled water, Wa is *W. anomalus*, Wa+C is *W. anomalus* cultivated with 1% chitosan, Bs is *B. subtilis*, and Sl is *S. liquefaciens*. The ratios correspond to the strains in the order shown in the figure after the CK (i.e., the first ratio for the first strain and the second ratio for the second strain). Different letters represent significant differences between the treatments according to Tukey’s mean comparison method at *p* < 0.05.

The antagonistic activities of *B. subtilis* (Bs), *W. anomalus* (Wa), *W. anomalus* incubated with 1% chitosan (Wa+C), *S. liquefaciens* (Sl), and their combinations against *P. expansum* was determined in vitro by measuring the growth of colony diameter on PDA. As it can be seen from Figure 2a–f, all strains individually or combined significantly reduced the fungal growth compared to the control (*p* < 0.05).

Individual application of Wa+C exhibited the strongest inhibition (reducing the lesion diameters by 55–70%), followed by Wa and Bs. Sl alone showed slightly weaker inhibition than the other individual applications but still better than the CK. Combinations of these strains at various ratios (1:1 to 1:4 and reverse) generally provided comparable or stronger efficacy to the individual antagonists.

The combined application of Bs and Wa at a ratio of 2:1 showed the best result than the individual application or the other combination ratios (Figure 2a). Notably, the combination of Wa+C and Bs at a 4:1 ratio demonstrated the best overall results, achieving significantly smaller colony diameters than the individual applications of Bs or Wa+C alone (Figure 2b). This suggests synergistic enhancement when Wa is chitosan-treated and applied in higher proportions with Bs. Across all the experiments, Wa+C consistently showed better performance than Wa, highlighting the role of chitosan in boosting yeast antagonism. The Sl-based combinations showed moderate effects similar to Sl alone (Figure 2c–e). Combined application of Wa with Wa+C at 4:1 and 3:1 ratios showed the same result but best among the other treatment groups (Figure 2f).

### 3.3. Determination of the Best Combination and Ratio to Inhibit the Growth of P. expansum In Vivo

**Figure 3:**
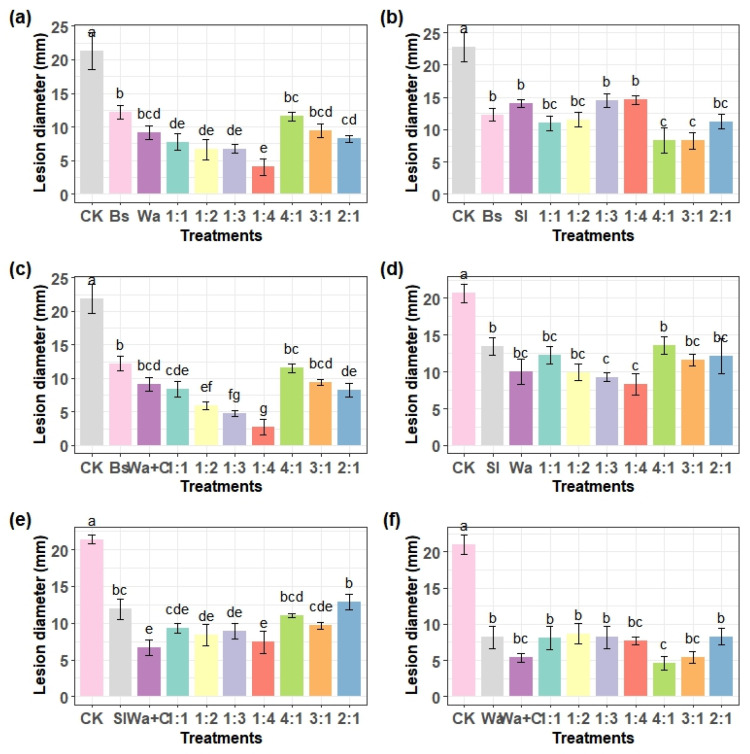
Determination of the effective combination by ratio of the antagonistic microbes that inhibit *P. expansum* growth in vitro after 4 days of storage at 28 °C. *B. subtilis* and *W. anomalus* (**a**), *S. iquefaciens* and *B. subtilis* (**b**), *B. subtilis* and *W. anomalus* incubated with chitosan (**c**), *S. iquefaciens* and *W. anomalus* (**d**), *S. iquefaciens* and *W. anomalus* incubated with chitosan (**e**), *W. anomalus* alone and *W. anomalus* incubated with chitosan (**f**). CK is double distilled water, Wa is *W. anomalus*, Wa+C is *W. anomalus* cultivated with 1% chitosan, Bs is *B. subtilis*, and Sl is *S. liquefaciens*. The ratios are as the strains shown in the figure order after the CK (i.e., the first ratio for the first strain and the second ratio for the second strain). Different letters represent significance difference between the treatments according to Tukey’s mean comparison method at *p* < 0.05.

Similar to the in vitro experiment, the efficacy of individual and combined biocontrol agents was evaluated against *P. expansum* on grapes by measuring the growth of lesion diameters. As can be seen from Figure 3a–f, the individual strain applications are still significantly better compared to the control (*p* < 0.05). The individual application of Wa+C showed the strongest inhibition (reducing lesion diameters by 40–65%), followed by Wa, and Bs (Figure 3a,c,f). The individual application of Sl exhibited moderate efficacy, similar to the in vitro experiment (Figure 3b,d,e).

Combined application of Bs and Wa at 1:4 ratio showed the best inhibitory effect and the combined application of Bs and S1 at 4:1 and 3:1 ratios showed the same result but exhibited better inhibitory effects than the other treatment groups (Figure 3a,b). Combined treatments at different ratios generally enhanced or matched the performance of individual agents. The combination of Wa+C and Bs at a 4:1 ratio showed higher inhibition efficiency than individual application (Figure 3c). This confirms the synergistic effect of the two antagonists. Similar to the in vitro results, the Sl-based combinations showed moderate effects (Figure 3d,e). Combination of Wa and Wa+C at the ratio of 4:1 demonstrated the best effect (Figure 3f). Since the combined application of Wa+C and Bs at the ratio of 4:1 by volume showed the best inhibitory results both in vitro and in vivo, we selected the two strains for the consecutive experiments.

### 3.4. Spore Germination and Germ Tube Length

**Figure 4:**
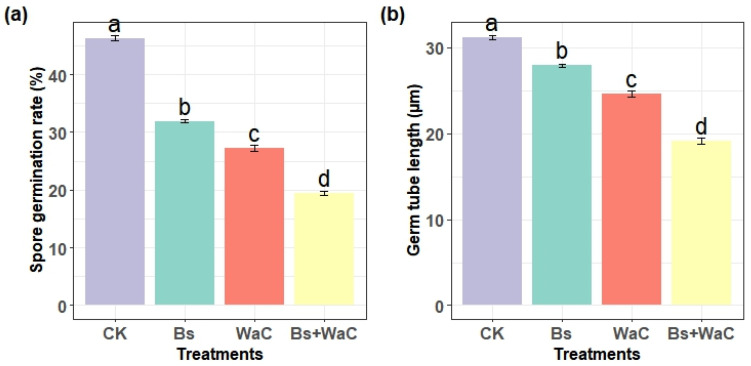
Effects of different treatment methods on the spore germination percentage (**a**) and germ tube length (**b**) of *P. expansum* after 10 and 12 h of incubation, respectively in PDB medium. Ck is control, Bs is *B. subtilis* alone, WaC is *W. anomalus* incubated with 1% chitosan alone, and Bs+WaC is *B. subtilis* plus *W. anomalus* incubated with 1% chitosan in a ratio of 1:4. The values are means ± standard deviations of at least three replications and different letters show significance difference (*p* < 0.05) according to Tukey’s HSD test.

The spore germination of individual and combined *B. subtilis* and *W. anomalus* incubated with chitosan was determined after 10 h of incubation in PDB. Compared to the control, which showed a germination rate of around 40%, all the three treatments significantly reduced spore germination (Figure 4a). *B. subtilis* alone decreased the rate to about 30%, while chitosan-enhanced *W. anomalus* lowered it to around 25%. The combined treatment of Bs and WaC demonstrated the strongest inhibition, reducing germination to roughly 15%, which was significantly lower than both individual treatments (Figure 4a).

Similarly, germ tube elongation was determined after 12 h of incubation in the PDB (Figure 4b). The control showed an average germ tube length of about 30 μm, whereas Bs alone reduced it to around 25 μm, and WaC alone to 20 μm, both representing significant decreases (*p* < 0.05). The Bs+WaC combination at 1:4 achieved the greatest suppression, limiting germ tube length to around 10 μm (Figure 4b).

### 3.5. Decay Rate and Lesion Diameter

**Figure 5:**
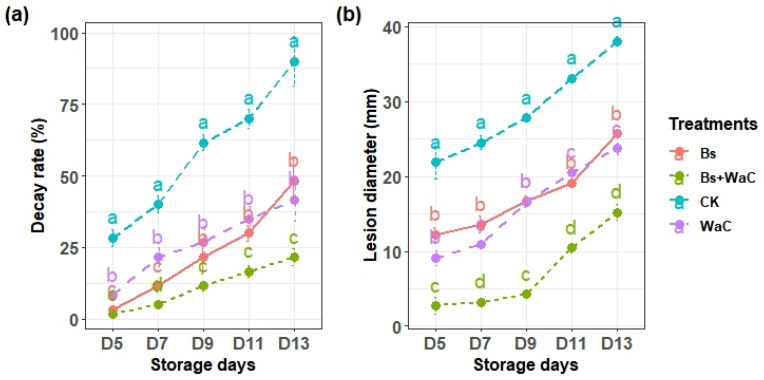
Effects of different treatment methods on the decay rate (**a**) and lesion diameter (**b**) of *P. expansum* during different table grapes’ storage period. Ck is control, Bs is *B. subtilis* alone, WaC is *W. anomalus* incubated with 1% chitosan alone, and Bs+WaC is *B. subtilis* plus *W. anomalus* incubated with 1% chitosan in a ratio of 1:4. The values are means ± standard deviations of at least three replications and different letters show significance differences (*p* < 0.05) according to Tukey’s HSD test.

The decay rate percentage and lesion diameter of *P. expansum* were measured for 13 days. In the control group, the decay percentage rapidly increased from 28% on day 5 to 90% on day 13, indicating rapid fungal invasion of the fruit. However, on grapes treated with *B. subtilis* and *W. anomalus* incubated with chitosan, the decay was less than 2% on day 5 and increased to around 21% on day 13 (Figure 5a). Both *B. subtilis* and *W. anomalus* incubated with chitosan alone showed better results than the control, but the combination showed the best control.

Lesion diameter also showed the same result during the storage period. The control group showed high lesion expansion from 21 mm on day 5 to 38 mm on day 13. *B. subtilis* alone and *W. anomalus* incubated with chitosan alone significantly inhibited the lesion growth of *P. expansum* compared to the control group (Figure 5b). Effective control was recorded for treatments with combined application of *B. subtilis* and *W. anomalus* at a 1:4 ratio. The average lesion growth of *P. expansum* was only 2.7 mm on day 5, and it was around 15 mm on day 13.

### 3.6. Effects on Natural Decay and Other Postharvest Quality Parameters of Grapes

The effects of biocontrol treatments on table grape postharvest physicochemical quality parameters were evaluated after seven days. As can be seen in Table 1, all three treatments significantly reduced the natural decay rate. The best result was obtained for grapes treated with *B. subtilis* plus *W. anomalus* incubated with chitosan (11.67%), followed by *B. subtilis* alone (21.67%), and then *W. anomalus* incubated with chitosan alone (26.67%). The decay percentage of the control sample was over 60%. Similarly, the lowest weight loss was recorded for grapes treated with *B. subtilis* plus *W. anomalus* incubated with chitosan (only 1.68%). There was no significant difference between the control samples and the other two treatments.

TTA and TSS showed minor variations between the treatments. The highest TTA and TSS were recorded for table grapes treated with *B. subtilis* alone. Table grape firmness was increased by all treatments relative to the control (3.73 N), with *B. subtilis* alone increasing it to 4.84 N, *W. anomalus* alone to 5.41 N, and *B. subtilis* plus *W. anomalus* showing the highest firmness (6.22 N). These results confirm that the treatments not only inhibit pathogen growth but also maintain desirable postharvest physicochemical attributes of table grapes.

### 3.7. Changes in the Enzymatic Activities of Table Grapes

**Figure 6:**
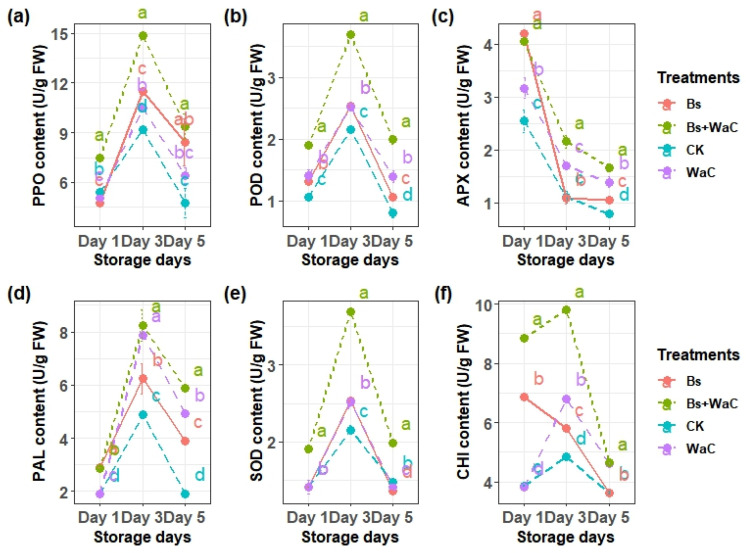
Changes in PPO (**a**), POD (**b**), APX (**c**), PAL (**d**), SOD (**e**), and CHI (**f**) enzymatic activities of table grapes stored at room temperature for five days. Ck is control, Bs is *B. subtilis* alone, WaC is *W. anomalus* incubated with 1% chitosan, and Bs+WaC is *B. subtilis* plus *W. anomalus* incubated with 1% chitosan in a ratio of 1:4. The values are means ± standard deviations of at least three replications and different letters within the storage days show significant differences between the treatments according to Tukey’s HSD test at *p* < 0.05.

Changes in the enzymatic activities of table grapes due to the biocontrol treatments over five storage days are shown in Figure 6. These disease defense and oxidative stress-related enzymes generally increased more due to the biocontrol treatments than in the control group. For example, PPO activity peaked at day 3 across all treatments and declined on day 5. The combination of *B. subtilis* and *W. anomalus* incubated with chitosan at a 1:4 ratio showed the best result (up to 15 U/g FW), while the control was only around 9 U/g FW (Figure 6a).

POD followed a similar pattern, peaking at day 3, where CK reached around 2.2 U/g FW, while treatments with *B. subtilis* plus *W. anomalus* again showed the highest enzymatic activity (3.7 U/g FW) (Figure 6b). The APX and SOD enzymes displayed antioxidant responses in the table grapes due to the biocontrol treatments. APX decreased overtime in all groups, but treatments with *B. subtilis* plus *W. anomalus* sustained significantly higher levels than the control group (Figure 6c).

Disease defense enzymes PAL and CHI also responded favorably to treatments. PAL activity reached its peak point on day 5 (more than 8 U/g FW) for treatments of *B. subtilis* plus *W. anomalus*, while the control group was under 5 U/g FW. The enzyme activity declined progressively on day 5, yet the biocontrol treatments preserved higher levels compared to the control group (Figure 6d). SOD showed the highest peak on day 3 and then declined on day 5. The treatments with *B. subtilis* plus *W. anomalus* were significantly higher than the other groups during the five storage days (Figure 6e). The CHI enzymatic activities of the *B. subtilis* plus *W. anomalus* peaked on day 3 and then declined on day 5. Treatments with *W. anomalus* alone also showed the same pattern. However, *B. subtilis* alone showed a decreasing trend during the whole storage period (Figure 6f). Overall, *B. subtilis* plus *W. anomalus* incubated with chitosan formulation at 1:4 demonstrated superior regulation of enzymatic profiles, mitigating oxidative damage and enhancing defense systems.

### 3.8. Relative Gene Expression

**Figure 7:**
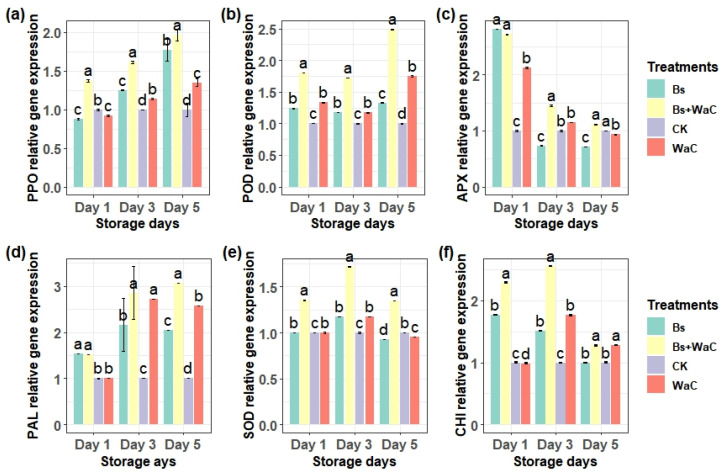
The relative gene expression of PPO (**a**), POD (**b**), APX (**c**), PAL (**d**), SOD (**e**), and CHI (**f**) key defense and oxidative stress related enzymes of table grapes stored at room temperature for five days. Ck is control, Bs is *B. subtilis* alone, WaC is *W. anomalus* incubated with 1% chitosan alone, and Bs+WaC is *B. subtilis* plus *W. anomalus* incubated with 1% chitosan in a ratio of 1:4. The values are means ± standard deviations of at least three replications and different letters within the storage days show significant differences between the treatments according to Tukey’s HSD test at *p* < 0.05.

The relative gene expression of key disease defense and oxidative stress-related enzymes in table grapes were analyzed using RT-qPCR over five storage days. The gene expressions of the enzymes were similar to the results of enzymatic activities, confirming the reliability of the previous results. As can be seen in Figure 7a–f, the results of most enzymatic activities were higher than those of the control samples, sometimes by up to 3-fold. The highest PPO relative gene expression was recorded for *B. subtilis* plus *W. anomalus* incubated with chitosan treatment on day five, which was almost 2-fold higher (Figure 7a). For POD the highest was also recorded on day five, which was around 2.5-fold (Figure 7b). *B. subtilis* alone and its combination with *W. anomalus* showed the highest APX gene expression on day one, which was near 2.8-fold of the control sample (Figure 7c). The highest PAL relative gene expression was recorded for *B. subtilis* plus *W. anomalus* incubated with chitosan treatment on day three and five, which was almost 3-fold that of the control (Figure 7d). SOD and CHI recorded the highest relative gene expressions compared to the control on day three for *B. subtilis* plus *W. anomalus* incubated with chitosan treatment, which were 1.7 times and 2.55 times higher, respectively (Figure 7e,f).

### 3.9. Total Phenols and Flavonoids Content

**Figure 8:**
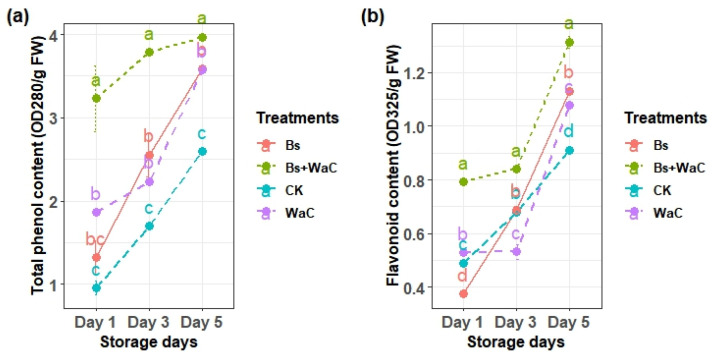
Changes in the total phenolic (**a**) and flavonoid (**b**) contents of table grapes during different storage periods. Ck is control, Bs is *B. subtilis* alone, WaC is *W. anomalus* incubated with 1% chitosan alone, and Bs+WaC is *B. subtilis* plus *W. anomalus* incubated with 1% chitosan in a ratio of 1:4. The values are means ± standard deviations of at least three replications and different letters within the storage days show significant difference between the treatments according to Tukey’s HSD test at *p* < 0.05.

Changes in the total phenol and flavonoid contents during the five storage days of table grapes are shown in Figure 8a,b. Both phenolic and flavonoid content increased over the storage time, reflecting the stress response to the *P. expansum* during the storage time. Treatments of the grapes with *B. subtilis* plus *W. anomalus* incubated with chitosan enhanced the accumulation of both compounds more than the other three samples during the whole time. Treatments with *B. subtilis* alone and *W. anomalus* alone increased the accumulation of these antimicrobial compounds better than the control samples.

### 3.10. Correlation Between Antifungal Compounds and Enzymes

**Figure 9:**
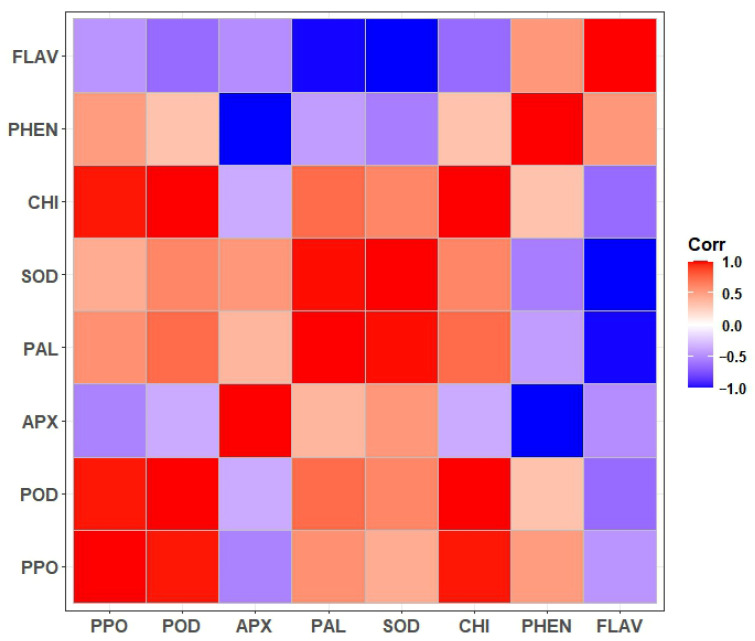
Correlation between disease defense-related enzyme activities and contents of antimicrobial compounds of table grapes treated with *B. subtilis* and *W. anomalus* incubated with chitosan. PPO (peroxidase activity), POD (polyphenol oxidase activity), APX (ascorbate peroxidase), PAL (phenylalanine ammonia lyase activity), SOD (superoxide activity), CHI (chitinase), PHEN (total phenol content), and FLAV (flavonoid content). The red color shows strong positive correlation, the blue color shows strong negative correlation, and the white color shows no correlation.

The correlation between disease defense-related enzymes and antifungal compounds is shown in Figure 9. As can be seen from the figure, the enzymes and the antifungal compounds have revealed complex positive and negative relationships. Strong positive correlations were observed among PPO, POD, SOD, and CHI. POD exhibited positive associations with PPO, PAL, SOD, CHI, and to a lesser extent PHEN. PPO and CHI showed positive associations with PHEN and moderate with FLAV. PHEN and FLAV also showed a positive correlation with each other. This indicates co-accumulation of key secondary metabolites. Conversely, negative correlations were observed for APX with PHEN and FLAV. A negative correlation was also shown for APX with PPO, POD, and CHI. PAL and SOD showed strong negative correlations with FLAV.

## 4. Discussion

Recent studies focus on either the individual application of antagonistic microbes by exploring new strains or enhancing the efficiency of the existing ones through various mechanisms to control postharvest diseases in fruits. Both *B. subtilis* and *W. anomalus* are well known for their antagonistic characteristics against postharvest fungal diseases [23,24,25]. But, when we examine all previous studies, the individual application of these antagonists alone is not equally competitive compared to the chemical pesticides. That is why the market share of biocontrol agents is still insignificant at the commercial scale.

In our previous studies, we determined that incubation of *W. anomalus* together with 1% chitosan for 24 h can increase its biocontrol efficiency against blue mold disease of table grapes by up to 10% [6,26]. Even if the yeast efficiency was improved (75% when applied alone vs. 85% when enhanced), for commercial and practical applications we still strive to improve the efficiency. Therefore, combined application of these two effective antagonists can be an alternative option to individual applications.

The present study demonstrates the synergistic potential of combining *B. subtilis* with chitosan-enhanced *W. anomalus* at a 1:4 ratio against postharvest blue mold disease of table grapes caused by *P. expansum*. This optimized formulation was selected from potential strains that might be combined to better inhibit the growth of *P. expansum*. *B. subtilis*, *W. anomalus* alone, *W. anomalus* incubated with chitosan, and *S. iquefaciens* were the candidates. With the affinity experiment, we confirmed that these strains do not mutually inhibit the growth of each other after 72 h at 28 °C. Therefore, their use in combined formulations to broaden the spectrum of pathogen control is feasible.

Among the combined strains, the combination of *B. subtilis* and *W. anomalus* incubated with chitosan at a 1:4 ratio showed the best inhibitory efficiency during the in vitro assays, with colony diameters significantly lower than those of the individual applications. The synergistic mechanism possibly involved complementary modes of action such as competition for nutrients, production of volatile compounds, and biofilm formation. A recent study by Guo et al. [26] demonstrated that *W. anomalus* induced with chitosan has better VOC production that inhibits fungal growth by disrupting membrane integrity and spore germination. Chitosan as an elicitor boosts the yeast metabolism, which increases the secretion of antifungal metabolites such as ethyl acetate and phenylethanol [27]. Furthermore, the inclusion of *B. subtilis*, known for its production of lipopeptides such as surfactin and iturin, complements the yeast action by directly lysing fungal hyphae [28]. This synergy of bacterial–yeast combinations brought the highest inhibition of *P. expansum* growth.

Similar to the in vivo experiment, the combined application reduced the lesion growth of *P. expansum* on grapefruit to 15 mm compared to the control, which was 35 mm. The decay rate was reduced to around 22%, while the control sample was 90% after 13 days of fruit storage. These results are better than the individual application of the two strains. This excellent performance might be due to the improved colonization of the two strains on the fruit surface in addition to the direct inhibition of the fungal growth. *W. anomalus*, when incubated with chitosan, showed better adhesion to the fruit surface and better film-forming properties [26,29]. Incorporating *B. subtilis* could potentially amplify more biofilm formation, better surface colonization, and adhesion as synergistic biocontrol.

The fruit physicochemical quality parameters were better preserved during the study period. The natural decay was just 11%, while the control was more than 60%. This is mainly due to delayed ripening and reduced ethylene production, facilitated by semi-coating by the antagonistic strains and microbial modulation of host metabolism. The weight loss (only 0.78%) was also better, which is a major factor for postharvest quality loss in fresh produce. The fruit texture was better retained in treatments of *B. subtilis* plus *W. anomalus*. The loss of firmness can lead to easy mechanical damage to the fruit during transport or storage. That can be an entry point for opportunistic pathogens as well. The TSS and TTA were maintained optimally, and there are no major differences between the treatment groups. Comparable results were obtained on strawberries and pears treated with yeast and bacterial strains [30,31].

The other underlying mechanism, in addition to direct inhibition of the pathogen growth, creating a physical barrier, and competition for nutrients and space, is inducing the fruit’s disease resistance. The combined application of *B. subtilis* and *W. anomalus* modulated the enzymatic activities of table grapes. Oxidative enzymes such as PPO and POD were elevated, which increases fruit resistance and reduces senescence by producing compounds such as lignin and melanin [31]. These compounds protect cells and delay spoilage by reducing oxidative stress, though PPO is linked to undesirable enzymatic browning if activated improperly [32].

The higher SOD and APX antioxidant enzymes in the treatment groups enhance ROS scavenging. SOD acts as the first line defense by converting highly reactive superoxide radicals (O_2_∙^−^) into hydrogen peroxide and molecular oxygen. After that, APX scavenges the H_2_O_2_ produced by SOD, reducing it to H_2_O by using ascorbate as an electron donor [33]. This reaction is central to the ascorbate glutathione cycle, which helps regulate cellular levels of H_2_O_2_ that could otherwise damage the fruit cells [34,35].

PAL and CHI enzymatic activities were also increased after the fruits treated with *B. subtilis* and *W. anomalus* were incubated in chitosan. The increment of these defense enzymes promotes the phenylpropanoid pathway and pathogen cell wall degradation. These are indicative of elicited systemic resistance. The same results were obtained in fruits treated with elicitors such as ursolic acid or melatonin, where enhanced PAL and CHI correlate with reduced fungal decay [36,37].

The contents of total phenols and flavonoids in treated fruits were better than the control and the individual applications throughout the five storage days. For example, the total phenol content of grapes treated with *B. subtilis* and *W. anomalus* incubated in chitosan was 3.95 units, while that of the control was only 2.62 units. The flavonoid content of the treatment group was 1.32 units while that of the control was 0.95 units on day five. These two act as antimicrobial and antioxidant agents and also strengthen the fruit cell walls and inhibit pathogen enzymes [38]. Correlation analysis showed strong positive correlation among PAL, SOD, CHI, and phenolics, highlighting integrated defense networks. For example, the role of PAL in phenolic biosynthesis positively correlates with CHI for fungal chitin degradation [39]. Negative correlations of APX with phenolics suggest balanced ROS signaling, preventing oxidative bursts while maintaining defense [40]. These correlations underscore how *B. subtilis* and *W. anomalus* organize a coordinated response, differing from individual treatments by amplifying synergistic pathways.

## 5. Conclusions

The current study successfully demonstrated that the combined application of *B. subtilis* and chitosan-enhanced *W. anomalus* at a 1:4 ratio better controlled blue mold disease of table grapes caused by *P. expansum*. While the individual applications also showed moderate inhibition in both in vitro and in vivo assays, the combined application surpassed the individual application. This synergy was developed through combined direct antagonism, competition for nutrients and space, and by inducing the grapes’ disease resistance. The antioxidant and disease defense-related enzymes and their relative gene expressions increased. The contents of antifungal compounds such as total phenol and flavonoids were elevated. The treatment increased the shelf life while preserving the physicochemical quality attributes of the grapes. This opens promising avenues for commercial biocontrol applications in the table grape industry, offering a sustainable, eco-friendly alternative to chemical fungicides amid increasing regulatory pressures and consumer preferences for residue-free produce. Future studies should focus on scaling up production, field trials under diverse climatic conditions, and multi-pathogen evaluations to validate broader applicability.

## Figures and Tables

**Table 1:** Effect of the combined applications of *W. anomalus* incubated with chitosan and *B. subtilis* on the postharvest physicochemical quality parameters of table grapes after seven storage days at room temperature.

Treatments	Physicochemical Quality Parameters				
	Natural Decay (%)	Weight Loss (%)	Titratable Acidity (%)	TSS (°Brix)	Firmness (N)
Ck	61.6667 ± 5.7735 ^a^	1.6789 ± 0.1618 ^a^	0.0020 ± 0.0001 ^c^	15.000 ± 0.000 ^b^	3.7286 ± 0.2042 ^d^
Bs	61.6667 ± 5.7735 ^a^	1.0967 ± 0.0270 ^b^	0.0032 ± 0.0001 ^a^	15.167 ± 0.057 ^a^	4.8374 ± 0.4168 ^c^
WaC	26.6667 ± 7.6376 ^b^	1.1220 ± 0.2025 ^b^	0.0027 ± 0.0001 ^b^	15.000 ± 0.000 ^b^	5.4081 ± 0.2571 ^b^
Bs + WaC	26.6667 ± 7.6376 ^b^	0.7778 ± 0.1138 ^b^	0.0019 ± 0.0001 ^c^	14.833 ± 0.057 ^c^	6.2249 ± 0.3749 ^a^

Ck is control, Bs is *B. subtilis* alone, WaC is *W. anomalus* incubated with 1% chitosan alone, and Bs+WaC is *B. subtilis* plus *W. anomalus* incubated with 1% chitosan in a ratio of 1:4. The values are means ± standard deviations of at least three replications and different letters within the column show significance differences (*p* < 0.05) according to Tukey’s HSD test.

## Data Availability

The raw data presented in this study and R codes used for data analysis and illustration can be available on request from the corresponding authors if the readers are interested.

## Abbreviations

The following abbreviations are used in this manuscript:

VOCs	Volatile organic compounds
NYDB	Nutrient yeast dextrose broth
NYDA	Nutrient yeast dextrose agar
Wa	*W. anomalus*
Wa+C	*W. anomalus* incubated with chitosan
Bs	*B. subtilis*
Sl	*S. liquefaciens*
PDA	Potato dextrose agar
PDB	Potato dextrose broth
PPO	Polyphenol oxidase
POD	Peroxidase
APX	Ascorbate peroxidase
PAL	Phenylalanine ammonia-lyase
SOD	Superoxide dismutase
CHI	Chitinase
